# Enhancing cultural competence in medical education

**DOI:** 10.5116/ijme.587a.0333

**Published:** 2017-01-26

**Authors:** Janne Sorensen, Marie Norredam, Nisha Dogra, Marie-Louise Essink-Bot, Jeanine Suurmond, Allan Krasnik

**Affiliations:** 1 Danish Research Centre for Migration, Ethnicity and Health, Department of Public Health, University of Copenhagen, Denmark; 2 Greenwood Institute of Child Health, Department of Neuroscience, Psychology and Behaviour, University of Leicester, Leicester, UK; 3 Academic Medical Center, University of Amsterdam, Department of Public Health, Amsterdam Public Health Research Institute, Amsterdam, The Netherlands

**Keywords:** Cultural competence, medical education, diversity sensitivity, medical curricula

## Introduction

A health system serving diverse populations requires health professionals who are competent in caring for patients and population groups who differ in e.g. age, gender, socio-economic status, migrant status, and ethnicity. Cultural competence (CC) among health professionals is viewed as one strategy to ensure equal access to healthcare across diverse groups and to ensure that patients receive care by their needs.^1^^,^^2^ However, many physicians are insufficiently prepared to meet the needs of increasingly diverse populations.^3^

In 2013, the EACEA ERASMUS Life Long Learning Programme funded the project Culturally Competent in Medical Education involving 13 partners from 11 countries.^4^ The project aimed to support the implementation of CC in medical curricula. First, a Delphi Study involving 34 experts was conducted to develop a framework of core cultural competencies for medical school teachers.  The framework included learning objectives on knowledge (e.g., teachers should have knowledge of determinants of health), attitudes (e.g., teachers should be aware of their own ethnic and cultural backgrounds), and skills (e.g., teachers should have the ability to engage and motivate all students).  The second stage of the project was a survey conducted to identify the strengths, gaps, and limitations of CC in the programmes of the 13 medical school project partners.

Based on the Delphi study and survey findings, we created guidelines for the development and delivery of CC training at medical schools.^4^ The proposed guidelines were presented in September 2015 in Amsterdam at a workshop entitled: “How to integrate cultural competence in medical education”. A range of participants attended the workshop, including the project partners, deans and faculty members of Dutch medical schools, physicians, and students, to test the relevance and clarity of the proposal. Based on the workshop discussions, some of the wording of the guidelines was modified, but the document remained substantially unchanged. The proposed guidelines are presented as minimum requirements; they generally concur with the previously published literature on the subject^5^^-^^7^ but cover a broader range of issues.

### Culturally competent teachers

Research shows that medical school teachers should be culturally competent to educate culturally competent physicians. Focus should, therefore, be placed on policies that recruit and build the capacity of culturally competent teachers. We suggest that the recruitment policies of the organisations ensure that teaching staff have the relevant qualifications and reflect the diversity of the community in which they work. This could be accomplished by explicitly stating in job advertisements for new staff that the recruiting organisation wishes to reflect the cultural diversity of society and therefore welcomes all qualified applicants. The advertisement should also state that if the applicant is not culturally competent, they should be willing to attend training in CC. This “condition” could be part of the formal contract. The advertisement should be distributed widely. 

We suggest that medical schools offer mandatory CC training for all teachers to enhance and strengthen teachers’ cultural competencies. It could be a specific CC course, or CC could be integrated into existing general teaching skills courses. Teachers’ CCs should also be evaluated, for example, by including questions about CC in student evaluations of the courses completed.

### Strengthening the curriculum

To strengthen the curriculum, there should be some minimum key CC learning objectives that should be covered, and students should be examined on these objectives. The content of the key competencies were defined in the Delphi study of the C2ME project and slightly adjusted in the assessment tool.^4^ They include “knowledge about a) key concepts including ‘culture’ and ‘ethnicity’, b) how social and cultural factors can affect health, health-related behaviours, and health care and c) key patient population groups to be identified in local sites (migration history, social conditions, specific health care needs, epidemiological data, risk factors, etc.)”.^4^ Another key competency is “attitude, defined as awareness of one’s implicit attitudes, including a) how one's own norms, values, and biases may affect health care provision, and b) how culture shapes individual behaviour and thinking (including the cultures of medicine)”.^4^ The last key competency is “skill, which is defined as the ability to a) work effectively with an interpreter and b) identify and take into account socio-cultural factors that may influence patient care (e.g., provide a treatment plan that takes into account the patient’s social and cultural context)”.^4^

CC can be included in the curriculum in several ways, including as a specific core mandatory component or by ensuring CC is integrated into the curriculum wherever relevant. Ensuring that course materials and literature reflect cultural diversity is also essential.

### Ensuring resources for activities and external advisors

Medical schools should earmark funding for developing CC courses for teachers, to ensure sufficient access to suitable teaching materials and tools and support from external advisors as needed. 

### Support from organisation and stakeholders

The success of integrating CC into the curriculum depends heavily on the support of those who develop institutional policies.  We, therefore, suggest that CC be included as an aim in the curriculum development and teaching strategies of the medical programme as well as in the overall medical school strategy, for example in the form of policies of inclusion. Examples of inclusive policies include those that challenge discrimination, bullying, harassment, and abuse, promote a safe and inclusive work and study environment and ensure that activities and festive traditions do not exclude any staff or students. Such initiatives will demonstrate to staff, students, and the wider community that the medical school and its stakeholders consider CC an important issue.

### Diversity of the student population

The diversity of the medical students’ ethnicities has been growing, which reflects the increasingly diverse populations of Europe. To ensure that the student population reflects the composition of the wider society on ethnicity, socio-economic status, gender, etc., student recruitment strategies should focus on groups underrepresented in medical schools in their country. The educational policies should also ensure that extracurricular activities facilitate student diversity. Finally, we suggest that students be evaluated after completing their studies to assess if they have acquired CC skills that adequately prepare them for future practice.

## Conclusions

At the level of individual patients, we know that migrant status and ethnicity interact with many characteristics such as age, gender, and socio-economic status to create unique beings, with unique challenges and resources. Health care organisations and professionals need to take this intersectionality into account. Therefore, attention to diversity issues should be considered during all stages of health care planning, including recruiting and training of health care staff and organising and providing health care.^8^^-^^9^ A key link in this chain of actions is ensuring CC in the medical profession. Medical schools should be the primary agents of change by taking the necessary steps in their institutional setup, curriculum development, and delivery of medical education. Medical schools could compare their programme with these guidelines to identify further actions to improve CC at their institution.

We understand that there are many competing priorities in the context of medical education. However, for CC to be achieved, it is essential to motivate and engage all relevant stakeholders and ensure diversity among the stakeholders. The process requires both a bottom-up and a top-down approach, where management takes responsibility, sets the strategy, and allocates resources, while faculty and students engage in suitable training and curriculum development. Such a dynamic process has the potential to broaden the experiences and competencies of those involved while also anticipating future challenges for healthcare and educational institutions, as European societies become even more diverse.

### Acknowledgements

We would like to acknowledge the C2ME partner institutions and research teams. The project Culturally Competent in Medical Education (C2ME) was funded by EACEA ERASMUS Life Long Learning Program.

### Conflict of Interest

The authors declare that they have no conflict of interest.
